# BRCA1 mutations in women with familial or early-onset breast cancer and BRCA2 mutations in familial cancer in Estonia

**DOI:** 10.1186/1897-4287-8-4

**Published:** 2010-04-09

**Authors:** Kristiina Tamboom, Krista Kaasik, Jelena Aršavskaja, Mare Tekkel, Aili Lilleorg, Peeter Padrik, Andres Metspalu, Toomas Veidebaum

**Affiliations:** 1 National Institute for Health Development, Tallinn, Estonia; 2 North Estonia Medical Centre, Diagnostics Division, Tallinn, Estonia; 3 Institute of Molecular and Cell Biology of the University of Tartu, Tartu, Estonia; 4 Estonian Biocentre, Tartu, Estonia; 5 The Estonian Genome Center of the University of Tartu, Tartu, Estonia; 6 Clinic of the Hematology and Oncology, Tartu University Hospital, Tartu, Estonia; 7 Department of Neurology, UCSF, CA 94158-2324, USA

## Abstract

**Background:**

The aim of this study was to identify BRCA1 and BRCA2 mutations in the Estonian population. We analyzed genetic data and questionnaire from 64 early-onset (< 45 y) breast cancer patients, 47 familial cases (patients with breast or ovarian cancer and a case of these cancers in the family), and 33 predictive cases (patients without breast or ovarian cancer, with a family history of such diseases) from Estonia for mutations in the BRCA1 gene. A sub-set of familial cases and predictive cases were also analyzed for mutations in the BRCA2 gene.

**Methods:**

For mutation detection, we used the Polymerase Chain Reaction-Single Stranded Conformation Polymorphism Heteroduplex Analysis (PCR-SSCP-HD), followed by direct DNA sequencing.

**Results:**

We identified three clinically important mutations in the BRCA1 gene, including seven occurrences of the c.5382insC mutation, three of c.4154delA, and one instance of c.3881_3882delGA. We also detected six polymorphisms: c.2430T>C, c.3232A>G, c.4158A>G, c.4427T>C, c.4956A>G, and c.5002T>C. Four sequence alterations were detected in introns: c.560+64delT, c.560+ [36-38delCTT, 52-63del12], c.666-58delT, and c.5396+60insGTATTCCACTCC. In the BRCA2 gene, two clinically important mutations were found: c.9610C>T and c.6631delTTAAATG. Additionally, two alterations (c.7049G>T and c.7069+80delTTAG) with unknown clinical significance were detected.

**Conclusions:**

In our dataset, the overall frequency of clinically important BRCA1 mutations in early-onset patients, familial cases, and predictive testing was 7.6% (144 cases, 11 mutation carriers). Pathogenic mutations were identified in 4 of the 64 early-onset breast cancer cases (6.3%). In familial cases, clinically important mutations in the BRCA1 gene were found in 6 of the 47 individuals analyzed (12.8%). In predictive cases, 1 clinically important mutation was detected in 33 individuals studied (3%). The occurrence of clinically important mutations in BRCA2 in familial cases of breast cancer was 2 of the 16 individuals analyzed (12.5%).

## Background

Breast cancer is the most common cancer in Estonian women between the ages of 30 and 74. Since 1978, the Estonian Cancer Registry has been collecting data regarding all cancer diagnoses in the country (population 1,370,052 in 2000). In the year 2000, 538 new cases of breast cancer were diagnosed, 65 of which occurred in women younger than 45. For all breast cancer cases, the crude incidence is 72.9 per 100,000 for Estonia [1]. Since the initial study localizing the breast cancer gene BRCA1 to chromosome 17q21 [2] and the isolation of the BRCA1 gene (OMIM#113705) [3], many mutation detection studies have been undertaken. The Breast Cancer Information Core (BIC) website http://research.nhgri.nih.gov/projects/bic/ and Cancer Genetics Web for BRCA1 describes more than 1500 mutations in the BRCA1 gene alone. The lifetime risk of breast cancer for Caucasian BRCA1 mutation carriers is 60-85%, while the lifetime risk of ovarian cancer is 15-40% [4]. The breast cancer susceptibility gene BRCA2 (OMIM#600185) [5] is also related to an increased risk of ovarian cancer [6].

The aim of the present study was to identify BRCA1 and BRCA2 mutations that could be used in diagnostics to provide the appropriate genetic counseling and treatment for patients in Estonia. We analyzed genetic data and questionnaire from individuals who had a family history of breast or ovarian cancer, as this is the major risk factor for familial breast cancer. In addition, developing breast cancer at a young age could indicate a genetic predisposition to the disease, particularly a mutation in the BRCA1 gene. Therefore, we also analyzed patients with an onset of breast cancer before the age of 45, regardless of family history. Because mutations may be localized throughout the BRCA1 and BRCA2 genes, the entire genes were analyzed in order to detect mutations in familial cases. Additionally, we analyzed the entire BRCA1 gene in early-onset cases. We used the Single Stranded Conformation Polymorphism Hederoduplex Analysis (SSCP-HD) method, followed by direct Sanger dideoxy DNA sequencing. The entire coding regions and exon-intron junctions in the BRCA1 and BRCA2 genes were sequenced in samples which presented abnormal electrophoresis patterns in SSCP-HD.

## Methods

### Patients

We screened for BRCA1 mutations in three groups of patients: early-onset, familial, and predictive cases, and also searched for BRCA2 mutations in all predictive and in some familial cases. The three patient groups are defined as the following:

1. Early-onset cases were identified as patients who had been diagnosed with breast cancer before age 45. We included patients who had been diagnosed during the five years prior to the start date of the study (Apr. 10, 2000), and also included new cases diagnosed after that date. Patient blood samples were collected between Apr. 10, 2000 and Feb. 10, 2003. A total of 95 patients were originally included in this group. Patients were asked to fill out questionnaires regarding relatives diagnosed with cancer. According to these questionnaires, 31 individuals reported additional breast or ovarian cancer cases in their family. These patients were removed from the early-onset group and were classified as familial cases. After taking into account cancer incidences in relatives of patients classified in the early-onset breast cancer group, 64 individuals remained.

2. The familial cases included in this study were individuals who had breast or ovarian cancer and at least one relative diagnosed with these cancers. Familial cases were identified at the Institute of Experimental and Clinical Medicine (present name National Institute for Health Development) and the Hematology and Oncology Clinic of Tartu University Hospital. Pedigrees were drawn according to information provided by the proband, and cancer diagnoses were confirmed in the Estonian Cancer Registry. Twenty-eight pedigrees encompassing 49 individuals blood samples and questionnaire were collected. Among these individuals, 17 had cancer (14 breast, 2 ovarian, and 1 cervical cancer), and the rest were high-risk relatives who were analyzed for predictive testing. Familial cases consisted of 16 patients with breast or ovarian cancer, who were analyzed for mutations in the BRCA1 and BRCA2 genes. Additional 31 patients diagnosed with early onset breast cancer who were found to have relatives with breast or ovarian cancer were also categorized as familial cases, and we analyzed the BRCA1 gene for mutations. In all, there were 47 familial cases.

3. Predictive testing cases included individuals from high risk families (with two or more relatives diagnosed with breast or ovarian cancer) who did not have breast or ovarian cancer themselves. This group consisted of 33 individuals.

Blood samples were collected at two oncology centers: the North Estonia Medical Centre's Centre of Oncology, and the Hematology and Oncology Clinic of Tartu University Hospital.

All individuals who participated signed informed consent forms. The study was approved by the Tallinn Medical Ethics Committee.

### Mutation screening

To detect BRCA1 and BRCA2 mutations, we used SSCP-HA followed by direct DNA sequencing. Genomic DNA was isolated from blood samples using a DNA Isolation Kit (Gentra Systems, Cat.no. GEND5500 or Fermentas UAB Cat. No. K0512). The Polymerase Chain Reaction (PCR) was used to amplify the entire coding sequence and splice junctions of the BRCA1 and BRCA2 genes. Intronic primers, designed by S. Gayther, were taken from the BIC Primer Database http://research.nhgri.nih.gov/projects/bic/ and used with minor modifications. The PCR reaction mixture contained PCR buffer, 1.5-3 mM MgCl_2_, 0.2 mM dNTPs, 10 pmol each primer, 2 U *Taq *DNA Polymerase, and 50-100 ng template DNA. The PCR program consisted of one cycle at 95°C for 1 min, followed by 28-35 cycles of: 95°C for 20 sec; 47-62°C (depending on the primers) for 30 sec; and 72°C for 1 min. For mutation screening, we used SSCP-HA. S. Gayther and B. Ponder describe the method in the BIC Methods Database http://research.nhgri.nih.gov/projects/bic/. SSCP and HA each have independent sensitivities between 60% and 80%. The use of these two techniques in combination (SSCP-HA) increases the overall sensitivity, because both analyses are performed on the same gel. We also used MDE (Mutation Detection Enhancement) gels for SSCP-HA analysis. MDE is a highly sensitive, high-resolution gel matrix specially formulated to separate DNA based on both size and conformation. This enables the detection of more mutations by SSCP-HA analysis than would be possible using standard polyacrylamide gels. The 0.6 × MDE (Cambrex, Cat.N. 50620) gels were analyzed by electrophoresis at 150-200V in TBE buffer. Electrophoresis was performed under two different conditions: one at 12°C and the other at 22°C. The DNA Silver Staining Kit (Amersham Pharmacia Biotech.Cat.N. 17-6000-30 or the method of Allen *et al*. [7]) was used for staining the gels. Fragments with aberrant mobility detected on SSCP-HA gels were sequenced with the BigDye Terminator cycle sequencing kit v3.1 (Applied Biosystems). DNA sequencing reactions were carried out on either ABI377 (Perkin Elmer) or ALF Express (Amersham Pharmacia Biotech.Cat.N. 56-1173-21) instruments. Both DNA strands were sequenced. All mutations were confirmed by repeat PCR and sequencing.

## Results

Thirteen sequence alterations were detected in the BRCA1 gene (Table 1), three of which were considered clinically significant, according to the BIC database: c.3881_3882delGA, c.4154delA, and c.5382insC. One of these, c.3881_3882delGA, was a novel mutation. The most frequent mutation in the BRCA1 gene was c.5382insC, of which seven cases were found. The c.4154delA mutation was detected in three cases. We also observed the following six polymorphisms: c.2430T>C, c.3232A>G, c.4158A>G, c.4427T>C, c.4956A>G and c.5002T>C. Four sequence rearrangements were detected in introns: c.560+64delT, c.560+ [36_38delCTT; 52_63del12], c.666-58delT, and c.5396+60insGTATTCCACTCC.

**Table 1:** Mutations, polymorphisms, synonymous substitutions, and alterations in introns in the BRCA1 and BRCA 2 genes from an Estonian population.

Mutation			Exon/intron	Type of mutation	Effect on amino acids	Early-onset N = 64	Familial N = 47	Predictive N = 33	Clinical importance in BIC
***BRCA1 Gene***									
**c.560+64delT**			7 intron	deletion		1	1	0	new
**c.560+ [36_38delCTT; 52_63delCTTTTTTTTTTT]**			7 intron	deletion		6	1	0	new
**c.666-58delT**			8 intron	deletion		1	1	0	new
**c.2430T>C**			11 exon	synonymous	Leu771Leu	3	6	1	no
**c.3232A>G**			11 exon	missense	Glu1038Gly	4	0	0	no
**c.3881-3882delGA**			11 exon	frameshift	stop 1265	0	1	0	yes
**c.4154delA**			11 exon	frameshift	stop 1365	0	3	0	yes
**c.4158A>G**			11 exon	missense	Arg1347Gly	0	0	3	unknown
**c.4427T>C**			13 exon	synonymous	Ser1436Ser	1	0	0	unknown
**c.4956 A>G**			16 exon	missense	Ser1613Gly	5	2	0	no
**c.5002T>C**			16 exon	missense	Met1628Thr	1	0	0	unknown
**c.5382insC**			20 exon	frameshift	stop 1829	4	2	1	yes
**c.5396+60insGTATTCCACTCC**			20 intron	duplication		1	0	0	unknown
***BRCA2 Gene***									
**c.6631delTTAAATG**			11 exon	deletion	Stop2167	0	1	0	new
**c.9610C>T**			25 exon	nonsense	Stop 3128	0	1	0	yes
**c.7049G>T**			11 exon	missense	Gly2274Val	0	2	0	unknown
**c.7069+80delTTAG**			11 intron	deletion		0	3	4	unknown

Four sequence alterations were identified in the BRCA2 gene (Table 1). Two of them were considered clinically important: c.9610C>T is considered relevant according to the BIC database, and c.6631delTTAAATG results in a stop in codon 2167. Two mutations of unknown clinical significance, c.7049G>T and c.7069+80delTTAG, were also observed.

A cohort of 95 women with early-onset breast cancer, diagnosed before age 45, was established and carefully analyzed for mutations in the BRCA1 gene. The average age of patients was 38 years, with the youngest patient being 24 years old. Early-onset breast cancer patients were also sub-grouped according to the incidence of cancer in relatives; patients who had at least one family member with breast or ovarian cancer, in addition to an early-onset diagnosis, were assigned as familial cases. Of the 95 patients, 31 had additional breast or ovarian cancer in the family and 64 patients without a familial history of these cancers were classified as early-onset cases. From these 64 early-onset patients, 4 (6.3%) carried the clinically important mutation c.5382insC in the BRCA1 gene. Twenty three early-onset patients had a mutation with unknown or no clinical significance in the BRCA1 gene (Table 1).

The 16 familial case patients who had breast or ovarian cancer and at least one relative with these cancers were analyzed for mutations in the BRCA1 and BRCA2 genes. In addition, 31 patients with early onset breast cancer and a family history of breast or ovarian cancer were analyzed for mutations in BRCA1. This group of familial cases contained a total of 47 patients. Among familial cases, mutations in the BRCA1 gene were observed in 6 patients among 47 individuals analyzed (12.8%). Taken together, these data indicate that the frequency of clinically important BRCA1 mutations in early-onset and familial cases in Estonia is 9% (10 mutations in 111 patients). With regard to the BRCA2 gene, only some familial cases (16 cases) and all predictive cases were analyzed, and 2 individuals from the familial group had clinically important mutations (12.5%). In predictive cases (N = 33), only one clinically important mutation was detected in the BRCA1 gene.

Estonia has a population of only 1.35 million people, and families tend to be quite small, with an average of 1.6 children per woman. Despite its small size, Estonia's population consists of several ethnic groups, the most common of which is Estonian (930,219), followed by Russian (351,178), Ukrainian (29,021) and Belorussian (17,241) [Statistics Estonia http://www.stat.ee/population-census-2000]. This distribution was reflected in the composition of our study participants. Our original early-onset breast cancer patient group consisted of 49 Estonians, 41 Russians, 2 Ukrainians, 2 Belorussians and 1 Latvian. Mutations were observed accordingly: 17 among Estonians, 19 in Russians, and 1 among both Ukrainians and Belorussians. In familial cases, nationality was not documented.

### Clinically important mutations in the BRCA1 gene

Three clinically important BRCA1 mutations were identified (Table 1 and 2). The c.5382insC mutation was the most frequent (63.6% of mutations), and was observed in four early-onset patients, two familial case patients, and one predictive case. The c.4154delA mutation is the second most frequent mutation, accounting for 27% of all mutations; it was found in three familial cases. We detected the mutation c.3881_3882delGA in one familial case (9% of mutations).

**Table 2:** Clinically important mutations in BRCA1 and BRCA2 genes and cancer incidence in relatives from Estonia.

Mutation	Patient ID	Nationality	Age of cancer diagnosis	Breast or ovarian cancer in relatives	Other cancers in relatives
***BRCA1 Gene***					
**c.3881_3882delGA**	fam. 72	Russian	44: BrCa	M:BrCa S:BrCa AU:BrCa	UN:LaCa
**c.4154delA**	fam. 37	Russian	40: BrCa	GM:OvCa	no
**c.4154delA**	fam. 13	Estonian	37: BrCa	M:BrCa	no
**c.4154delA**	fam. 14/A079	Russian	48: OvCa	M:OvCa	no
**c.5382insC**	early.40	Estonian	36: BrCa	no	no
**c.5382insC**	early. 49	Russian	39: BrCa	no	no
**c.5382insC**	early. 59	Russian	30: BrCa	no	GF1, GF2:GaCa, NI1:UtCa, UN:CoCa, NI2: Mel
**c.5382insC**	fam. 62	Russian	43: BrCa	M:BrCa	GM:GaCa, UN:LuCa
**c.5382insC**	early. 66	Russian	35: BrCa	no	no
**c.5382insC**	fam. 77	Russian	38: BrCa	M:OvCa	no
**c.5382insC**	pred. 49/A062	unknown	ND	S:BrCa, S:BrCa, AU:BrCa	no
***BRCA2 Gene***					
**c.6631delTTAAATG**	fam. 16/A106	unknown	65: OvCa	S: BrCa, AU:OvCa	UN:GaCa, B:PrCa,
**c.9610C>T**	fam. 03/A065	unknown	36: BrCa	M:BrCa, S:BrCa, NI:BrCa	no

Abbreviations:

fam. Familial cases, early. Early-onset case, pred. Predictive case, ND not diagnosed (predictive testing). M mother, S sister, B brother, GM grandmother, GF grandfather, AU aunt, UN uncle, NI niece. BrCa breast cancer, OvCa ovary cancer, LaCa larynx cancer, GaCa gastric cancer, UtCa uterus cancer, CoCa colon cancer, Mel melanoma, LuCa lung cancer, PrCa prostate.

### Sequence variations in the BRCA1 gene

Several sequence variations, which were not considered clinically important, were also observed. Six of these were detected in introns: the c.2430T>C variant (3 early-onset cases, 6 familial cases, and one predictive case), the c.3232A>G variant (4 early-onset cases), the c.4158A>G variant (3 predictive cases), the c.4427T>C variant (1 early-onset case), the c.4956A>G variant (5 early-onset cases and 2 familial cases), and the c.5002T>C variant (1 early-onset case) (Table 1). Four sequence alterations were detected in introns: c.560+64delT, c.560+ [36_38delCTT; 52_63del12], c.666-58delT, and c.5396+60insGTATTCCACTCC. The c.560+64delT and c.666-58delT alterations were both detected in 2 patients: one with early-onset breast cancer and one with familial cancer. The variant c.560+ [36_38delCTT; 52_63del12] was found in 7 patients, 6 with early-onset breast cancer and 1 with familial cancer. None of these mutations are described in the BIC database. Finally, c.5396+60insGTATTCCACTCC was detected in one patient with early-onset breast cancer. This mutation is described in the BIC database, but is considered to be a mutation of unknown clinical importance.

### Mutations in the BRCA2 gene

In familial cases, we found two clinically important mutations: c.6631delTTAAATG and c.9610C>T. The c.6631delTTAAATG mutation was detected in one individual, and is a novel mutation according to the BIC database. The 9610C>T mutation was detected in one individual. We also found two alterations with unknown clinical significance: c.7049G>T in two individuals with familial cancer, and c.7069+80delTTAG in three patients with familial cancer and four predictive cases.

### Incidence of cancer in relatives of BRCA1 and BRCA2 mutation carriers

We next analyzed the incidence of cancer in relatives of those individuals identified as carrying BRCA1or BRCA2 mutations. The cancer incidence in relatives is divided into two groups 1) breast or ovarian, and 2) other cancer types (Table 2). In the BRCA1 gene, four c.5382insC mutation carriers were found in early-onset cases: 3 of them did not have a family history of cancer, one patient's grandfathers had gastric cancer, uncle had colorectal cancer, nieces had melanoma and uterus cancer. Two c.5382insC mutation carriers were found in familial cases: one patient's mother had breast cancer and grandmother had gastric cancer, and uncle had lung cancer, and another patient's mother had ovarian cancer. One c.5382insC mutation carrier was observed among the predictive cases, three cases of breast cancer occurred in this family: two sisters and one aunt. Three c.4154delA mutation carriers were found among familial cases: one patient's grandmother had ovarian cancer, the second patient's mother had breast cancer, and the third family there was an additional case of ovarian cancer. One patient was a c.3881-3882delGA mutation carrier: her mother, sister, and aunt all had breast cancer, and her uncle had larynx cancer.

Among mutations in the BRCA2 gene, one c.6631delTTAAATG mutation carrier had a sister with breast cancer, her aunt had ovarian cancer, and in addition, her brother had prostate cancer and her uncle had gastric cancer. One individual with the 9610C>T mutation had three cases of breast cancer in her family: in her mother, sister and niece (Table 2).

## Discussion

The relatively small population and family size in Estonia is one reason families with at least two familial cases of breast or ovarian cancer were included. This is also the main reason we observed BRCA1 mutations in 12.8% and BRCA2 mutations in 12.5% of the individuals who were selected on the basis of family history. In early-onset breast cancer, pathogenic mutations in BRCA1 were found in 4 cases of 64 patients analyzed (6.3%). That falls within the range reported in other studies. For example, in a population-based study from France, the authors analyzed patients that had been diagnosed with breast cancer before age 46, and found deleterious mutations in 6.5% [8]. According to a population-based study from Sweden, 6.8% of breast cancer patients younger than 41 carry mutations in the BRCA1 gene [9]. The BRCA1 mutation carrier frequency in young breast cancer patients is lower in Estonia than in either Latvia or Russia, which lie just across its border. In Latvia, 26% of young breast cancer patients (diagnosed before age 48) were found to carry mutations in BRCA1 [10]. In a Russian study, breast cancer cases were selected according to the presence of clinical indicators of hereditary disease, such as bilaterality, onset by age 40, or family history, and breast-cancer-associated alleles were detected in 15.2% of subjects [11].

### Clinically important mutations in the BRCA1 gene

The most frequent BRCA1 mutation in Estonia is c.5382insC (63.6% of mutations). This mutation is highly prevalent among the Ashkenazi Jewish population and even more so in Eastern Europe. The c.5382insC mutation is thought to have originated in the Baltic region [[12] and [13]]. The frequency of this mutation is high in Central and Eastern Europe: Russia (94% - 96%) [[14] and [11]], Latvia (60%) [10] and Poland (51%) [15]. The c.5382insC mutation is also found in Finland [16]. The results from Estonia also support the theory regarding the origin of the c.5382insC mutation.

The second most frequent mutation in Estonia is c.4154delA (27% of mutations). This mutation is considered to be a specific mutation for Central and Eastern Europe [17].

In Latvia, which neighbors Estonia, the two most prevalent mutations, c.4154delA and c.5382insC, represent 80% of all BRCA1 mutations [10]. In Estonia, these mutations are also the most common c.5382insC (63.6%) and 4154delA (27%). The c.4154delA mutation is also prevalent in Finland [16].

The c.3881_3882delGA mutation was named according to nomenclature for the description of sequence variations [Human Genome Variation Society http://www.hgvs.org/mutnomen]. The same mutation with a different name, c.3880delAG, has been described in one breast cancer patient from Italy [18]. It appears in the Breast Cancer Information Core database once, and can be considered a rare mutation. This nonsense mutation generates a premature stop codon at codon 1265, and so c.3881_3882delGA can therefore be considered clinically important.

### Sequence alterations in the BRCA1 gene

The alterations in BRCA1 are considered not to be clinically important according to the BIC database and information in literature. Durocher and colleagues studied the allelic variants c.2430T>C, c.3232A>G, c.4427T>C, and c.4956A>G, and found no statistically significant difference in allele frequency between breast and ovarian cancer patients compared to a control population [19]. Deffenbaugh and colleagues found that the variant c.4158A>G occurs at a heterozygote frequency of 2%. The corresponding R1347 is not conserved in the murine BRCA1 protein, and does not lie within the functional domains of BRCA1. For these reasons, they interpreted the c.4158A>G as a polymorphism being of no substantial clinical significance [20]. Phelan and his group noted that if an unknown variant co-occurs with a known deleterious mutation, it is unlikely to confer high risk itself. An example of this is c.5002T>C (M1628T). In addition, a functional assay of the M1628T variant showed transcriptional activation similar to or greater than the wild type BRCA1. Together, these data suggest that c.5002T>C is not clinically significant [21].

### Mutations in the BRCA2 gene

Among familial cases, we found two clinically important mutations: c.6631delTTAAATG and c.9610C>T. The c.6631delTTAAATG mutation is new according to the BIC database. It is considered to be clinically important because the mutation results in a stop codon at codon 2167.

## Conclusions

We analyzed DNA from a total of 144 individuals from Estonia: 64 women with early-onset breast cancer diagnosed before the age of 45, and 47 familial cases consisting of patients with breast or ovarian cancer and at least one family member diagnosed with these cancers, and 33 predictive cases, individuals without breast or ovarian cancer, but having at least two cases of these cancers in the family; performing a screen for mutations in the BRCA1 gene. A sub-set of familial cases (N = 16) and all predictive cases were additionally analyzed for mutations in the BRCA2 gene. We used SSCP-HA followed by DNA sequencing. Pathogenic mutations were identified in 4 of the 64 early-onset breast cancer cases (6.3%). In familial cases, clinically important mutations in the BRCA1 gene were found in 6 of the 47 individuals analyzed (12.8%). In predictive cases, 1 clinically important mutation was detected in 33 individuals studied (3%). The occurrence of clinically important mutations in BRCA2 in familial cases of breast cancer was 2 of the 16 individuals analyzed (12.5%). We identified three clinically important mutations in the BRCA1 gene in the cohort. The most frequent mutation was c.5382insC (63.6%), the second most frequent was c.4154delA (27%), and we identified one individual with the c.3881_3882delGA mutation (9%). We also observed several common polymorphisms in addition to three new and one known sequence alteration in introns. In familial cases, we identified two clinically important mutations in the BRCA2 gene, c.6631delTTAAATG and c.9610C>T (both in one case), and two mutations of unknown clinical significance: c.7049G>T and c.7069+80delTTAG. The mutation pattern of BRCA1 from Estonia is characteristic for Eastern and Central Europe.

## Competing interests

The authors declare that they have no competing interests.

## Authors' contributions

KT participated in PCR, SSCP-HD and sequencing, participated in the sequence alignment and wrote the manuscript. KK participated in the design of the study, participated in PCR, SSCP-HD and sequencing, participated in the sequence alignment. JA participated in PCR, SSCP-HD and sequencing. MT participated in the design of the study and helped to draft the manuscript. AL participated in the design of the study. PP helped to draft the manuscript. AM conceived of the study, and participated in its design and coordination and helped to draft the manuscript. TV participated in the design of the study and helped to draft the manuscript.

All authors read and approved the final manuscript.
